# The Safety and Effectiveness of Apixaban in Patients with End-Stage Kidney Disease on Dialysis: A Retrospective Observational Study

**DOI:** 10.3390/jcm13051351

**Published:** 2024-02-27

**Authors:** Wasim El Nekidy, Emna Abidi, Said Nabil, Saba Kendakji, Moatasem Ali, Salahdein Aburuz, Bassam Atallah, Fadi Hijazi, Jihad Mallat, Amal Akour

**Affiliations:** 1 Department of Pharmacy Services, Cleveland Clinic Abu Dhabi, Abu Dhabi P.O. Box 112412, United Arab Emirates; abidiee@clevelandclinicabudhabi.ae (E.A.); atallab@clevelandclinicabudhabi.ae (B.A.); 2 Cleveland Clinic Lerner College of Medicine, Case Western Reserve University, Cleveland, OH 44106, USAmallatj@clevelandclinicabudhabi.ae (J.M.); 3 Department of Pharmacology and Therapeutics, College of Medicine and Health Sciences, United Arab Emirates University, Al Ain P.O. Box 15551, United Arab Emirates; 700036435@uaeu.ac.ae (S.N.); 202170017@uaeu.ac.ae (S.K.); 700036432@uaeu.ac.ae (M.A.); saburuz@uaeu.ac.ae (S.A.); 4 Critical Care Institute, Cleveland Clinic Abu Dhabi, Abu Dhabi P.O. Box 112412, United Arab Emirates; 5 Department of Biopharmaceutics and Clinical Pharmacy, School of Pharmacy, The University of Jordan, Amman 11942, Jordan

**Keywords:** direct acting oral anticoagulant, end stage renal diseases, dialysis

## Abstract

**Background:** Apixaban has been increasingly utilized for various FDA-approved indications, including stroke prevention and venous thromboembolism (VTE) treatment in patients with end stage kidney disease (ESKD) on hemodialysis. However, the safety and efficacy of its use in this population is not well established. Hence, the purpose of this study is to evaluate the safety and effectiveness of apixaban by examining outcomes in this population. **Methods:** This was a retrospective observational study that involved adults with ESKD who were on hemodialysis and prescribed apixaban from our hospital’s outpatient pharmacy between 1 May 2015, and 31 March 2022. Demographics, apixaban indications, dose appropriateness, concomitant antiplatelet use, and comorbidities data were collected. Bleeding and thromboembolic events were also collected. **Results:** Sixty-six patients fulfilled the inclusion criteria, 50% of them males. Median age was 71 (63.5–82) years, and the median BMI 28.2 (59.5–86.25) kg/m^2^. The median follow-up time was 5 (1.9–12.3) months. Concomitant antiplatelet use (39.4%) and high medication adherence (84.8%) were observed. During follow-up, major bleeding events occurred in 15.2% of cases, with minor bleeding being more common (36.4%), and VTE and stroke events occurred in 4.5% of cases; appropriate dosing was prevalent (62.1%), and there was an overall all-cause mortality rate of 34.8%. Most patients received a 2.5 mg BID apixaban dose (56.1%), including both NVAF and VTE groups. Notably, the multivariate logistic regression analysis indicated that weight, and daily dose were insignificant predictors of bleeding events (*p* = 0.104, 0.591), however, the BMI was the main independent risk factor for bleeding in this population [OR = 0.9, 95% CI: 0.8–0.99; *p* = 0.023]. **Conclusions:** Our analysis of apixaban-treated ESKD patients highlights that the risk of bleeding is significant, and BMI was the main independent risk factor. A larger prospective study is needed to confirm our findings.

## 1. Introduction

The use of direct oral anticoagulants (DOACs) is steadily increasing for various Food and Drug Administration (FDA)-approved indications, including stroke prevention in patients with non-valvular atrial fibrillation (NVAF), as well as the prophylaxis/treatment of venous thromboembolism (VTE) [1]. Patients with chronic kidney disease (CKD) are at a particularly high risk for VTE [2] and stroke [3], regardless of the degree of renal impairment, but this is especially aggravated in patients with end stage kidney disease (ESKD) who are on dialysis. In addition, there is an increased risk of bleeding in this vulnerable population, which might be a result of renal disease complications such as anemia, thrombocytopenia, and secondary hyperparathyroidism, or concomitant medications, including anticoagulants and antiplatelets, or the coexistence of certain coagulopathies [4]. Furthermore, the hemodialysis process imposes an inherent risk of bleeding, due to creation of vascular access points such as arteriovenous fistula or grafts [5]. Two DOACs, rivaroxaban and apixaban, are FDA-approved for the above-mentioned indications in patients with ESKD on hemodialysis [6]. A very recent (2021) systematic review showed that, compared to warfarin, apixaban did not significantly decrease the incidence of VTE recurrence in ESKD patients on dialysis, however, fewer major bleeding events were reported in apixaban-treated patients [7]. For stroke prevention in patients with NVAF, another systematic review showed that, although apixaban (used in a standard dose of 5 mg twice daily) reduced the risk of stroke and systemic embolism compared to warfarin, this dose led to higher rates of bleeding [8]. In addition, rivaroxaban use was associated with a high rate of related bleeding and mortality [8]. Although a lower-than-standard dose of DOACs for stroke prevention was associated with less bleeding in dialysis patients, their efficacy remains controversial, as the available observational data on apixaban/rivaroxaban need to be confirmed on a larger scale, and many studies did not systematically evaluate the appropriateness of DOAC therapy. To date, the compelling evidence to support the use of DOACs for various indications (i.e., VTE prophylaxis/treatment and stroke prevention in NVAF) in ESKD patients on dialysis is still debatable. Therefore, the main aim of this study is to evaluate the safety and effectiveness of apixaban and the appropriateness of apixaban prescription, correlating the latter with related safety and efficacy outcomes in this population.

## 2. Methodology

### 2.1. Study Design and Settings

This was a retrospective observational study utilizing a convenience sample of patients with ESKD on dialysis who were receiving apixaban in an outpatient pharmacy setting, at our quaternary care hospital, between 1 May 2015, and 31 March 2022. Patients were included if they were adults (≥18 years), had ESKD treated with intermittent hemodialysis (IHD) three times weekly, and were receiving apixaban. Patients with incomplete data, or who had received apixaban for less than one month, or who were not established on IHD (<3 months) were excluded. Patients’ demographics, baseline characteristics, pertinent laboratory findings, apixaban doses, and therapy durations were all collected. In addition, concomitant medications (e.g., antiplatelets), and bleeding events were also documented. Furthermore, dialysis-related data, CHA2DS2-VASc score, and any previous venous thromboembolic events were also documented. The procedures used in this study adhered to the tenets of the Declaration of Helsinki. Informed consent was waived by the CCAD REC, in view of the retrospective nature of the present study and the fact that all the procedures performed were part of the patient’s routine care.

### 2.2. Study Outcomes

The primary outcome assessed was the safety of apixaban and, specifically, bleeding incidents. The secondary outcomes assessed were venous thromboembolic events or strokes, as well as dose appropriateness. Variables were defined as described in the following.

The bleeding events were documented as either a major or a minor bleed, according to the International Society on Thrombosis and Hemostasis (ISTH) criteria [9] and included significant gastric bleeding, hemorrhagic stroke, and life-threatening bleeding. The secondary outcomes included factors that are related to dose appropriateness and bleeding risk. The effectiveness of apixaban was measured by the occurrence/recurrence of VTE and/or the rate of ischemic/embolic stroke in NVAF patients, as well as safety.

Apixaban appropriate dose was evaluated against the FDA-approved indication, which is 5 mg twice daily in VTE (following an acute period of treatment of 7 days with 10 mg BID or parenteral anticoagulation), and 5 mg BID in atrial fibrillation unless patient weight is <60 kg and age is >80 years in addition to being on dialysis; in that case, the dose should be reduced to 2.5 mg BID.

### 2.3. Statistical Analysis

Data normality and distribution were assessed using the Shapiro–Wilk test and by visually inspecting the variable’s distribution (histogram). Data are expressed in terms of median (IQR). Proportions were used as descriptive statistics for categorical variables. The Mann–Whiney U test was used to perform a comparison of values between independent and continuous variables, while discrete data comparisons were performed by Chi-Square or Fisher’s exact test when the numbers were small. The independent risk factors of bleeding were assessed using multivariate logistic regression analysis. Clinically relevant variables were assessed with univariate analysis, including variables that are known to affect the bleeding risk. Goodness-of-fit was assessed using the Hosmer–Lemeshow test. A two-sided *p*-value < 0.05 was used as the criterion to determine statistical significance in this study. The variables assumed a 2-sided significance level of 0.05. All statistical analyses were performed using the SPSS statistical package, version 29 for Microsoft Windows (IBM, Armonk, NY, USA).

## 3. Results

A total of 66 patients met the inclusion criteria. Males represented 50% of the sample, and the sample as a whole had a median age of 71 (63.5–82) years, a median weight of 70.5 (59.5–86.25) Kg, and a median body mass index (BMI) of 28.2 (59.5–86.25) kg/m^2^. Baseline characteristics and demographics are described in Table 1. The major indication for apixaban was stroke prevention in NVAF patients, with a median duration of apixaban follow-ups of 5 (1.9–12.3) months, and an adherence rate of 84.8% (n = 56). Most patients received a 5 mg total daily apixaban dose (56.1%, n = 37) including both NVAF and VTE cases. Appropriate apixaban dosing was reported in 62.1% of subjects (n = 41).

At follow-up, major bleeding events occurred in 15.2% (n = 10) and minor bleeding in 36.4% (n = 24) of the studied patients. VTE and stroke events occurred in 4.5% of cases, with no statistical differences in the bleeding vs. non-bleeding groups (*p* = 0.293 and 1, respectively), and the all-cause mortality rate was 34.8% (n = 23) (Table 2). When baseline characteristics were stratified by bleeding, no significant differences were found between bleeding vs. non-bleeding arms (Table 1). Prevalent comorbidities, including diabetes and dyslipidemia, showed no significant differences between bleeding and non-bleeding groups (Table 2).

The multivariate logistic regression analysis indicated that BMI is the main independent risk factor for bleeding in this population [OR = 0.9; 95%CI (0.8–0.99); *p* = 0.023]; therefore, patients with lower BMIs had more bleeding risk. It also indicated that both age and apixaban daily dose were insignificant predictors of bleeding events. When weight was added to the regression model instead of BMI, it was shown that the weight is not a significant independent predictor for bleeding (Table 3). The most common major bleeding types that occurred with those with lower BMI were rectal bleeding, hemoptysis, upper GI bleed, and multiple site bleeding.

We further stratify data by appropriateness (Table S1). Baseline demographics showed that there were significantly more patients with stroke in the group with appropriate dosages (n = 7, 17.1% vs. n = 1, 4%; *p*-value = 0.045). The median apixaban daily dose was significantly higher with appropriate apixaban [10 (5–10) vs. 5 (5–7.5), *p*-value = 0.011]. The rest of variables had no statistically significant differences.

## 4. Discussion

To our knowledge, this is the first study in our region to address the appropriateness of apixaban therapy and the associated determinants of bleeding in patients with ESKD on dialysis. Importantly, this is the first study to show that lower BMI is associated with higher risk of bleeding, as compared to just weight. Furthermore, the values for concomitant antiplatelet therapy were not statistically different between bleeding and non-bleeding groups. Appropriate apixaban dosing was observed in 62.1% of patients, and was associated with a high adherence rate. However, bleeding, predominantly minor, occurred at a high rate in this group of patients, compared to those using low and high doses (Table S2). On the other hand, apixaban was effective in preventing VTE and stroke recurrence in 95.5% of the studied patients, regardless of bleeding. All-cause mortality occurred in more than one-third of the patients. While the use of DOACs for prevention of stroke in NVAF patients with a CrCl < 15 mL/min is contraindicated according to the European guidelines, the US FDA has previously stated that insufficient data exists to assess apixaban use in patients with CrCl < 15 m/min or who are on dialysis. Therefore, the lack of consensus requires caution in prescribing this medication for this specific patient population [10]. Most studies have evaluated the safety and effectiveness outcomes in patients with CKD who are not on dialysis, and only few studies have included only ESKD patients on dialysis. A retrospective cohort study that was performed on a total of 25,523 US patients, including 2351 dialysis patients on apixaban, reported that, comparing apixaban to warfarin in matched cohorts, there were no differences in the risks of stroke and systemic embolism; specifically, there was a 12.4 vs. 11.8 event rate per 100 patients’ years for apixaban and warfarin, respectively (HR 0.88, 95% CI 0.69–1.12; *p* = 0.29). Apixaban caused a significantly lower risk of major bleeding, with 19.7 event rates per 100 patient years vs. 22.9 in the warfarin-treated group (HR 0.72, 95% CI 0.59–0.87; *p* < 0.001) [8]. Additionally, a study by Ellenbogen et al. that compared 2302 apixaban patients to 9263 warfarin ESKD patients on hemodialysis or peritoneal dialysis demonstrated a lower risk of major bleeding, 10.3% vs. 13.7% (HR 0.81, 95% confidence interval [CI]: 0.70–0.94), as well as total clinically relevant nonmajor bleeding, 15.3 vs. 18.1 (HR 0.84, 95% CI: 0.74–0.94); intracranial, 1.8 vs. 2.5 (HR 0.69, 95% CI 0.48–0.98); and gastrointestinal bleeding, 8.6 vs. 10.4 (HR 0.82, 95% CI 0.69–0.96), respectively [11]. When compared to our results, the study showed fewer major bleeding events, as well as considerably fewer minor bleeding events (15.2% and 36.4%, respectively). Similarly, the rates of recurrent VTE events were higher than those observed in our cohort, at 6.6 vs. 4.5%. However, the rate of all-cause mortality was lower than in our study, specifically, 10 vs. 34.8% [11]. Furthermore, a retrospective cohort study by Reed D et al., also confirmed that the apixaban-treated group (n = 74) had fewer overall bleeding events when compared to their warfarin-treated counterparts (n = 50) (18.9% vs. 42.0%; *p* = 0.01). Additionally, the apixaban-treated group experienced fewer frequent major bleeding events than did patients in the warfarin group (5.4% vs. 22.0%; *p* = 0.01), while both groups experienced no recurrent ischemic strokes. The same study also highlighted less recurrent VTE incidence in patients on apixaban, compared with those on warfarin (4.4% vs. 28.6%; *p* = 0.99); however, these results remain statistically non-significant [12]. Overall, the above-described results suggest that apixaban can be used as a safe and effective alternative to warfarin in patients with ESRD on dialysis.

Furthermore, a systematic review and meta-analysis conducted by El Far et al. which included five large clinical studies, out of which three involved apixaban, evaluated the efficacy and safety of DOACs (n = 3044), as compared to warfarin (n = 31,472), in patients with NVAF on dialysis [13]; it showed a cumulative bleeding risk and mortality rate lower than those observed in our study (12.2% and 11.4%, respectively). Another small study by Sarrat et al. which included 40 ESKD patients on chronic dialysis, although with no reported duration of apixaban use, showed a favorable risk profile for apixaban, with no major bleeding events [14].

In addition, the meta-analysis conducted by El Far et al. included five studies, with a total of 31,472 (92.14%) patients on warfarin and 3044 (8.91%) patients on DOACs, and showed no significant differences in the incidences of hemorrhagic stroke, major bleeding, hemodialysis access site bleeding, ischemic stroke, or gastrointestinal bleeding between DOACs and warfarin-treated patients. However, patients who received DOACs presented with higher rates of systemic embolization, minor bleeding, and death events than was reported in the warfarin group ((3.39% vs. 1.97%, *p*-value = 0.02), (6.78% vs. 2.2%, *p*-value 0.02), and (11.38% vs. 5.12%, *p*-value < 0.006), respectively) [13]. Welander et al. also showed that in a total of 2453 Swedish patients with NVAF and CKD G3-G5D, the DOAC-treated group (41%) showed a lower hazard of major bleeding [HR 0.71 (95% confidence interval 0.53–0.96)], but there was no difference in ischemic stroke risk, compared with the warfarin group (59%). However, mortality was higher during DOAC treatment [1.24 (1.01–1.53)], although this was presumably not a causal association, since fewer fatal bleedings occurred on DOAC [15]. Accordingly, it is pertinent to highlight that, despite the agreement between different existing definitions of major bleeding, such as those by the ISTH and the Bleeding Academic Research Consortium, which can be similarly interpreted to evaluate patients’ prognostics, some other definitions, such as the Thrombolysis in Myocardial Infarction criteria, have shown discrepancies in identifying patients with the highest clinical risks [16]. Therefore, anticoagulation therapy decisions should, in general, take into consideration relevant criteria that could predictively and independently characterize clinically relevant morbidity as well as death risks [17]. Furthermore, it is well established that warfarin is eliminated mainly via the liver, but reduced dosing is mandatory in the dialysis patient to decrease the risk of bleeding [18]. The dosing of DOACs has also been discussed in a review conducted by Vio R et al., where a standard dose of apixaban, defined as (5 mg twice a day; n = 1034), significantly reduced the risks of stroke, systemic embolism, and death, as compared to either a lower dose of apixaban (2.5 mg twice a day; n = 1317; HR 0.61, 95% CI 0.37–0.98, *p* = 0.04 for stroke/systemic embolism; and HR 0.64, 95% CI 0.45–0.92, *p* = 0.01 for death) or to warfarin (HR 0.64, 95% CI 0.42–0.97, *p* = 0.04 for stroke/systemic embolism; and HR 0.63, 95% CI 0.46–0.85, *p* = 0.003 for death) [19].

Moreover, in a systematic review, multiple studies confirmed a lower risk of major bleeding with apixaban, in ESKD patients on dialysis, as compared to warfarin. The apixaban doses in those studies ranged between 2.5 mg BID to 10 mg BID [7]. Furthermore, in a multi-center retrospective chart review, Hanni et al. performed a head-to-head comparison of the 2.5 mg BID (n = 57) vs. the 5 mg BID (n = 77) dosage. Interestingly, patients on apixaban 5 mg BID had a longer lag-time to bleed and a lower rate of bleeding (0.0% for 5 mg BID, 9.5% for 2.5 mg BID, and 10.5% for warfarin, *p* = 0.04), with no difference found in thrombosis (*p* = 0.374) [20]. In our study, dose was shown to be an insignificant predictor of bleeding events (*p* = 0.104, 0.591), for both NVAF and VTE, in ESKD patients on dialysis.

As a general note about the dosing of DOACs, a previous study conducted by our team has concluded that inappropriate underdosing of DOACs has been deemed common in the region, particularly with apixaban and rivaroxaban, a phenomenon which also has been associated with higher risks of stroke and bleeding [21], rendering further follow-up, and more specific evaluations of the dosing of DOACs, in ESKD patients on dialysis worth performing on a larger as well as a multi-center scale. Overall, our findings did not match many of those studies. It is unclear whether the opposite findings in our study were due to the regional population, which was different from those of the listed studies. However, it is of pertinence to highlight that our analysis of apixaban-treated patients showed a positive correlation, with low BMI as a main independent risk factor for bleeding, in our population of ESKD patients on dialysis [OR = 0.9, 95% CI:0.8–0.99; *p* = 0.023]. Additionally, a multi-center study by Novak et al. aimed to look at the safety and efficacy outcomes of DOACs (n = 11,604) vs. those of warfarin (n = 8093), in normal and extreme-weight patients, with 295 patients being underweight and 9108 patients being pre-obese to obese class 3. The study’s results showed that obese patients were 24.6% more likely to experience a composite outcome of thromboembolism, symptomatic recurrent VTE, or severe bleeding, when compared to normal BMI patients. Moreover, underweight patients were more likely to be diagnosed with a bleed, compared to normal BMI patients (OR 1.522, 95% CI 1.095–2.115), while obese patients showed less bleed occurrence (OR 0.749, 95% CI 0.658–0.854) [22]. Also, in accordance with our findings, a retrospective comparison of the effect of BMI on the safety and efficacy of DOACs (n = 3458) showed increased-bleeding rates to be inversely proportional to increases in BMI [23]. However, Barakat et al. showed that DOACs are associated with better safety and effectiveness across all BMI categories, in 36,094 NVAF patients, including underweight and morbidly obese patients [24]. Therefore, the effects of DOACs across patients of different BMI categories have been largely discussed, but to our knowledge, this is the first study showing results in a specific cohort of apixaban-treated ESKD patients on dialysis.

It is noteworthy to recognize that anticoagulants have a critical role in preventing thrombosis of arteriovenous fistulas (AVF) in hemodialysis patients. AVF have been associated with immediate and midterm elevated all-cause and cardiovascular mortality risks, particularly within the first 90 days post-event, and when access restoration is delayed beyond 7 days [5]. Therefore, future research to explore the role of DOACs in mitigating arteriovenous fistula thrombosis is warranted.

Our study has, also, some limitations. The retrospective study design could introduce bias because of confounders, the relatively small sample size, and the lack of a comparative arm with other anticoagulants, including warfarin. Future studies should include larger cohorts within prospective designs and compare these outcomes to other standard therapies, e.g., warfarin. In the current study, the CHADS2-VASC score was utilized as a predictive clinical measure for estimating the risk of stroke in NVAF patients. On the other hand, the risk of major bleeding was not evaluated. The HAS-BLED (i.e., Hypertension (HTN), Abnormal Renal/Liver Function, Stroke, Bleeding History, Labile INR, Elderly, and Drugs/Alcohol) score, which is used to assess the benefits vs. the risks of adding anticoagulants to a therapeutic regimen, could have been used [25]. Still, in hemodialysis patients, the utility of this score has still to be further elucidated, as these patients are inherently at a higher risk of bleeding due to uremic homeopathy and the frequent use of heparin [26]. A 2019 systematic review has shown that this score, compared to other bleeding scores, has a low predictive value in dialysis patients, and thus it is of little clinical utility [27].

So far, there has been shown no high-quality evidence to support using DOACs in dialysis patients. Therefore, the current guidelines still give weak recommendations for using them in patients with NVAF on dialysis [28]. The hypothesis of this study posited that apixaban would effectively mitigate VTE and stroke recurrences in end stage kidney disease (ESKD) patients undergoing dialysis. The study’s conclusion indicates that apixaban was associated with reduced risks of both stroke recurrence and VTE. However, it also highlights that in patients with low BMI, there is a statistically significant increase in the risk of minor bleeding, a finding which warrants careful consideration and further investigation in a larger cohort of patients.

## Figures and Tables

**Table 1:** Baseline characteristics of patients by bleeding groups.

Baseline Characteristics			Total (n = 66)	No Bleeding (n = 32)	Bleeding (n = 34)	*p*-Value
Males n (%)			33 (50)	14 (43.8)	19 (55.9)	0.325
Median Age (IQR)years			71 (63.5–82)	68.5 (59.5–76.5)	75.5 (66.8–83.3)	0.039
Median Weight (IQR) kg			70.5 (59.5–86.25)	71.0 (60.0–88.0)	66.5 (55.3–79.0)	0.12
BMI (Kg/m^2^)	N (%)	>25	37 (56.1)	21 (65.6)	16 (47.1)	0.21
		18–24.9	24 (36.4)	10 (31.3)	14 (41.2)	
		<18	5 (7.6)	1(3.1)	4 (11.8)	
	Median BMI (IQR)		28.3 (22.6–32.8)	29.9 (23.9–34.1)	24.8 (20.7–30.3)	0.037
Apixaban Daily Dose	n (%)	5 mg	37 (56.1)	17 (53.1)	20 (58.8)	0.64
		10 mg	29 (43.9)	15 (46.9)	14 (41.2)	
	Median (IQR)		5 (5–10)	-	-	0.66
DVT/PE n (%)			13 (19.7)	6 (18.8)	7 (20.6)	0.85
Atrial Fibrillation n (%)			54 (81.8)	26 (81.3)	28 (82.4)	0.91
Diabetes n (%)			60 (90.9)	31(96.9)	29 (85.3)	0.20
Dyslipidemia n (%)			60 (92.3)	30 (96.8)	30 (88.2)	0.36
Stent n (%)			33 (50)	15 (46.9)	18 (52.9)	0.81
CABG Within 12 Months n (%)			19 (28.8)	11(34.4)	8 (23.5)	0.33
Liver Disease n (%)			14 (21.2)	7 (21.9)	7 (20.6)	0.90
Malignancy n (%)			4 (6.1)	2 (6.3)	2 (5.9)	1.00
Hypertension n (%)			60 (90.9)	30 (93.8)	30 (88.2)	0.673
CAD/ACS Within 12 Months n (%)			19 (28.8)	11 (34.4)	8 (23.5)	0.41
LV Thrombus n (%)			3 (4.5)	1 (3.1)	2 (5.9)	1.00
Smoking n (%)			16 (24.2)	6 (18.8)	10 (29.4)	0.31
Heart Failure n (%) (HFrEF, and HFpEF)			47 (71.2)	23 (71.9)	24 (70.6)	0.91
Aortic Valve Stenosis n (%)		Mild	11 (16.7)	7 (21.9)	4 (11.8)	0.18
		Moderate	5 (7.6)	4 (12.5)	1(2.9)	
		Severe	5 (7.6)	1 (3.1)	4 (11.8)	
LVAD n (%)			4 (6.1)	0 (0)	4 (11.8)	0.11
Stroke n (%)		Ischemic	7 (10.6)	2 (6.3)	5 (14.7)	
		Hemorrhagic	1 (1.5)	1 (3.1)	0 (0)	0.33
Concomitant Antiplatelet n (%)		1 Antiplatelet	26 (39.4)	9 (28.1)	17(50)	0.14
		2 Antiplatelets	16 (24.2)	8 (25)	8 (23.5)	
Adherent (no more than 30 days between refills) n (%)			56 (84.8)	26 (81.3)	30 (88.2)	0.51
Duration With Medication (months) Median (IQR)			5 (1.9–12.3)	3.5 (1.1–8.6)	5 (2.0–14.8)	0.22

BMI: body mass index; CAD/ACS: coronary artery disease/acute coronary syndrome; CABG: coronary artery bypass graft; DVT/PE: deep vein thrombosis/pulmonary embolism; LV: left ventricular; LVAD: left ventricular assist device. HFrEF and HFpEF: heart failure with reduced ejection fraction and preserved ejection fraction; respectively.

**Table 2:** Outcomes at follow-up in all patients, stratified by bleeding status.

Outcomes		Total (n = 66)	No Bleeding (n = 32)	Bleeding (n = 34)	*p*-Value
Dose appropriateness n (%)		41(62.1)	21 (65.6)	20 (58.8)	0.57
Dose appropriateness n (%)	Low	19 (28.8)	9 (28.1)	10 (29.4)	0.71
	Appropriate	41(62.1)	21 (65.6)	20 (58.8)	
	High	6 (9.1)	2 (6.3)	4 (11.8)	
DVT/PE during follow-up n (%)		3 (4.5)	0 (0)	3 (8.8)	0.29
Stroke during follow-up n (%)		3 (4.5)	1(3.1)	2 (5.9)	1.00
Mortality n (%)		23 (34.8)	9 (28.1)	14 (41.2)	0.27
INR Median (IQR)		1.3 (1.1–1.5)	1.3 (1.1–1.5)	1.25 (1.1–1.5)	0.87
CHA2DS2_VASc Score Median (IQR)		5.0 (4.0–6.0)	5.0 (4.0–6.0)	5 (4.0–6.3)	0.59

CHA2DS2_VASc: categories included Congestive heart failure, Hypertension, Age ≥ 75 years (doubled), Diabetes mellitus, prior Stroke or TIA or thromboembolism (doubled), Vascular disease, Age 65 to 74 years, and Sex. DVT/PE: deep vein thrombosis/pulmonary embolism. INR: international normalized ratio.

**Table 3:** Multiple logistic regression of factors related to bleeding risk.

	B	Odds Ratio (95% Confidence Interval)	*p*-Value
Apixaban daily dose (mg)	0.061	1.06 (0.85–1.33)	0.591
Age (Years)	0.036	1.04 (0.99–1.08)	0.104
BMI (Kg/m^2^)	−0.103	0.9 (0.8–0.99)	0.023

## Data Availability

Data are available on request from the authors.

## Supplementary Materials

The following supporting information can be downloaded at: https://www.mdpi.com/article/10.3390/jcm13051351/s1, Table S1: Analysis Based on Dose Appropriateness; Table S2: Outcome stratified by dose appropriateness.

## References

[1] Chen A., Stecker E., Warden B.A. (2020). Direct Oral Anticoagulant Use: A Practical Guide to Common Clinical Challenges. J. Am. Heart Assoc..

[2] Christiansen C.F., Schmidt M., Lamberg A.L., Horváth-Puhó E., Baron J.A., Jespersen B., Sørensen H.T. (2014). Kidney disease and risk of venous thromboembolism: A nationwide population-based case-control study. J. Thromb. Haemost..

[3] Kelly D.M., Ademi Z., Doehner W., Lip G.Y.H., Mark P., Toyoda K., Wong C.X., Sarnak M., Cheung M., Herzog C.A. (2021). Chronic Kidney Disease and Cerebrovascular Disease: Consensus and Guidance From a KDIGO Controversies Conference. Stroke.

[4] Van Eck Van der Sluijs A., Abrahams A.C., Rookmaaker M.B., Verhaar M.C., Bos W.J.W., Blankestijn P.J., Dekker F.W., Van Diepen M., Ocak G. (2021). Bleeding risk of haemodialysis and peritoneal dialysis patients. Nephrol. Dial. Transplant..

[5] Girerd S., Girerd N., Frimat L., Holdaas H., Jardine A.G., Schmieder R.E., Fellström B., Settembre N., Malikov S., Rossignol P. (2020). Arteriovenous fistula thrombosis is associated with increased all-cause and cardiovascular mortality in haemodialysis patients from the AURORA trial. Clin. Kidney J..

[6] Alhousani M., Malik S.U., Abu-Hashyeh A., Poznanski N.J., Al-Hasan S., Roth D.F., Alsharedi M., Mustafa B. (2021). Using oral anticoagulants among chronic kidney disease patients to prevent recurrent venous thromboembolism: A systematic review and meta-analysis. Thromb. Res..

[7] Cheung C.Y.S., Parikh J., Farrell A., Lefebvre M., Summa-Sorgini C., Battistella M. (2021). Direct Oral Anticoagulant Use in Chronic Kidney Disease and Dialysis Patients With Venous Thromboembolism: A Systematic Review of Thrombosis and Bleeding Outcomes. Ann. Pharmacother..

[8] Siontis K.C., Zhang X., Eckard A., Bhave N., Schaubel D.E., He K., Tilea A., Stack A.G., Balkrishnan R., Yao X. (2018). Outcomes Associated With Apixaban Use in Patients With End-Stage Kidney Disease and Atrial Fibrillation in the United States. Circulation.

[9] Chen H.-Y., Ou S.-H., Huang C.-W., Lee P.-T., Chou K.-J., Lin P.-C., Su Y.-C. (2021). Efficacy and Safety of Direct Oral Anticoagulants vs Warfarin in Patients with Chronic Kidney Disease and Dialysis Patients: A Systematic Review and Meta-Analysis. Clin. Drug Investig..

[10] Deal E.N., Pope H., Ross W. (2014). Apixaban Use Among Patients With Severe Renal Impairment. Ann. Pharmacother..

[11] Ellenbogen M.I., Ardeshirrouhanifard S., Segal J.B., Streiff M.B., Deitelzweig S.B., Brotman D.J. (2022). Safety and effectiveness of apixaban versus warfarin for acute venous thromboembolism in patients with end-stage kidney disease: A national cohort study. J. Hosp. Med..

[12] Reed D., Palkimas S., Hockman R., Abraham S., Le T., Maitland H. (2018). Safety and effectiveness of apixaban compared to warfarin in dialysis patients. Res. Pract. Thromb. Haemost..

[13] Elfar S., Elzeiny S.M., Ismail H., Makkeyah Y., Ibrahim M. (2022). Direct Oral Anticoagulants vs. Warfarin in Hemodialysis Patients With Atrial Fibrillation: A Systematic Review and Meta-Analysis. Front. Cardiovasc. Med..

[14] Sarratt S.C., Nesbit R., Moye R. (2017). Safety Outcomes of Apixaban Compared With Warfarin in Patients With End-Stage Renal Disease. Ann. Pharmacother..

[15] Welander F., Renlund H., Dimény E., Holmberg H., Själander A. (2023). Direct oral anticoagulants versus warfarin in patients with non-valvular atrial fibrillation and CKD G3–G5D. Clin. Kidney J..

[16] Xu Y., Gomes T., Wells P.S., Pequeno P., Johnson A., Sholzberg M. (2022). Evaluation of definitions for oral anticoagulant-associated major bleeding: A population-based cohort study. Thromb. Res..

[17] Chan K.E., Giugliano R.P., Patel M.R., Abramson S., Jardine M., Zhao S., Perkovic V., Maddux F.W., Piccini J.P. (2016). Nonvitamin K Anticoagulant Agents in Patients With Advanced Chronic Kidney Disease or on Dialysis With AF. J. Am. Coll. Cardiol..

[18] Alshogran O.Y. (2019). Warfarin Dosing and Outcomes in Chronic Kidney Disease: A Closer Look at Warfarin Disposition. CDM.

[19] Vio R., Proietti R., Rigato M., Calò L.A. (2021). Clinical Evidence for the Choice of the Direct Oral Anticoagulant in Patients with Atrial Fibrillation According to Creatinine Clearance. Pharmaceuticals.

[20] Hanni C., Petrovitch E., Ali M., Gibson W., Giuliano C., Holzhausen J., Makowski C., Pallisco A., Patel N., Sutter D. (2020). Outcomes associated with apixaban vs warfarin in patients with renal dysfunction. Blood Adv..

[21] Anouassi Z., Atallah B., Alsoud L.O., El Nekidy W., Al Mahmeed W., AlJaabari M., Almuti K. (2021). Appropriateness of the Direct Oral Anticoagulants Dosing in the Middle East Gulf Region. J. Cardiovasc. Pharmacol..

[22] Novak A.R., Shakowski C., Trujillo T.C., Wright G.C., Mueller S.W., Kiser T.H. (2022). Evaluation of safety and efficacy outcomes of direct oral anticoagulants versus warfarin in normal and extreme body weights for the treatment of atrial fibrillation or venous thromboembolism. J. Thromb. Thrombolysis.

[23] Netley J., Howard K., Wilson W. (2019). Effects of body mass index on the safety and effectiveness of direct oral anticoagulants: A retrospective review. J. Thromb. Thrombolysis.

[24] Barakat A.F., Jain S., Masri A., Alkukhun L., Senussi M., Sezer A., Wang Y., Thoma F., Bhonsale A., Saba S. (2021). Outcomes of Direct Oral Anticoagulants in Atrial Fibrillation Patients Across Different Body Mass Index Categories. JACC Clin. Electrophysiol..

[25] Pisters R., Lane D.A., Nieuwlaat R., De Vos C.B., Crijns H.J.G.M., Lip G.Y.H. (2010). A Novel User-Friendly Score (HAS-BLED) To Assess 1-Year Risk of Major Bleeding in Patients With Atrial Fibrillation. Chest.

[26] Murphy M., Maddox W., Nahman S., Diamond M., Sorrentino R., Guha A., Waller J. (2015). Abstract 12014: Modified HASBLED Bleeding Risk Score in Dialysis Patients With Atrial Fibrillation. Circulation.

[27] Ocak G., Ramspek C., Rookmaaker M.B., Blankestijn P.J., Verhaar M.C., Bos W.J.W., Dekker F.W., Van Diepen M. (2019). Performance of bleeding risk scores in dialysis patients. Nephrol. Dial. Transplant..

[28] January C.T., Wann L.S., Calkins H., Chen L.Y., Cigarroa J.E., Cleveland J.C., Ellinor P.T., Ezekowitz M.D., Field M.E., Furie K.L. (2019). 2019 AHA/ACC/HRS Focused Update of the 2014 AHA/ACC/HRS Guideline for the Management of Patients With Atrial Fibrillation. J. Am. Coll. Cardiol..

