# Supplementary material for: The Safety and Effectiveness of Apixaban in Patients with End-Stage Kidney Disease on Dialysis: A Retrospective Observational Study
Source: J Clin Med. 2024 Feb 27;13(5):1351. doi: 10.3390/jcm13051351 (PMC10931560; doi:10.3390/jcm13051351)
Supplement: Supplementary file 1 [file jcm-13-01351-s001.zip › jcm-2882878-supplementary.pdf]

Supplementary file S1

Supplementary Table S1: Analysis Based on Dose Appropriateness:

**Baseline Characteristics**		**Frequency**			**P-Value**
		**Inappropriate dose**	**Appropriate dose**	**Total**	
**Gender**	Male	13(52%)	20(48.8%)	33(50%)	1
	Female	12(48%)	21(51.2%)	33(50%)	
**BMI (Kg/m^2)**	>25	10(40%)	27(65.9%)	37(56.1%)	0.102
	18-24.9	13(52%)	11(26.8%)	24(36.4%)	
	<18	2(8%)	3(7.3%)	5(7.6%)	
**DVT/PE**		7(28%)	6(14.6%)	13(19.7%)	0.214
**Atrial Fibrillation**		18(72%)	36(87.8%)	54(81.8%)	0.186
**Diabetes**		23(92%)	37(90.2%)	60(90.9%)	1
**Dyslipidemia**		22(88%)	38(95%)	60(92.3%)	0.365
**Stent**		15(60%)	18(43.9%)	33(50%)	0.31
**CABG within 12 months**		9(36%)	10(24.4%)	19(28.8%)	0.403
**Liver disease**		7(28%)	7(17.1%)	14(21.2%)	0.358
**Cancer**		2(8%)	2(4.9%)	4(6.1%)	0.63
**Hypertension**		24(96%)	36(87.8%)	60(90.9%)	0.396
**CAD/ACS within 12 months**		8(32%)	11(26.8%)	19(28.8%)	0.653
**LV thrombus**		3(12%)	0(0%)	3(4.5%)	0.05
**Smoking**		6(24%)	10(24.4%)	16(24.2%)	1
**Congestive Heart Failure**		19(76%)	28(68.3%)	47(71.2%)	0.583
**Sleep apnea**		10(40%)	17(41.5%)	27(40.9%)	1
**Mechanical valve**		1(4%)	2(4.9%)	3(4.5%)	1
**Hypothyroidism**		9(36%)	5(12.2%)	14(21.2%)	0.022
**Aortic Valve Stenosis**	Mild	6(24%)	5(12.2%)	11(16.7%)	0.177
	Moderate	3(12%)	2(4.9%)	5(7.6%)	
	Severe	3(12%)	2(4.9%)	5(7.6%)	
**LVAD**		2(8%)	2(4.9%)	4(6.1%)	0.63
**PVD**		1(4%)	8(19.5%)	9(13.6%)	0.137
**PAD**		4(16%)	8(19.5%)	12(18.2%)	1
**Stroke**	Ischemic	0(0%)	7(17.1%)	7(10.6%)	0.045*
	Hemorrhagic	1(4%)	0(0%)	1(1.5%)	
**Concomitant antiplatelet**	1 antiplatelet	14(56%)	12(29.3%)	26(39.4%)	0.056
	2 antiplatelet	6(24%)	10(24.4%)	16(24.2%)	
**Adherent (no more than 30 days between refills)**		20(80%)	36(87.8%)	56(84.8%)	0.485
**Switching for another anticoagulant**		5(20%)	4(9.8%)	9(13.6%)	0.282

Variable	**Percentiles**		**P- Value**
	**Inappropriate dose**	**Appropriate dose**	
**Apixaban Daily Dose**	5(5-7.5)	10(5-10)	0.011
**Age**	71(63.5-78)	71(62.5-83)	0.937
**Weight**	65(57-84)	71(60-88)	0.272

**BMI (Kg/m^2)**	24.219(21.74-30.79)	29.29(24.39-33.78)	0.087
**Duration with medication**	6(2.5-15)	3.0(1.0-8.5)	0.112
**Ejection Fraction %**	56(35.5-65)	57.0(41.5-64.0)	0.995
**Total Cholesterol mmol/l**	3.195(2.68-3.79)	3.165(2.43-3.65)	0.547
**HDL-C mmol/l**	0.85(0.71-1.102)	1.04(0.84-1.24)	0.064
**LDL-C mmol/l**	1.75(1.35-2.30)	1.4(1.12-2.18)	0.367
**INR**	1.2(1.0-1.4.0)	1.3(1.12-1.60)	0.015
**CHA2DS2_VASc Score**	6.0(4.0-7.0)	5.0(4.0-6.0)	0.203

CHA2DS2_VASc: Congestive heart failure, Hypertension, Age ≥75 years (doubled), Diabetes mellitus, prior Stroke or TIA or thromboembolism (doubled), Vascular disease, Age 65 to 74 years, Sex category. DVT/PE: deep vein thrombosis/pulmonary embolism; INR: international normalized ratio; LV: Left ventricular. BMI: body mass index; CAD/ACS: coronary artery disease/acute coronary syndrome; CABG: coronary artery bypass graft; DVT/PE: deep vein thrombosis/pulmonary embolism; LV: Left ventricular; LVAD: left ventricular assist device; PVD: peripheral vascular disease' PAD: peripheral arterial disease. HDL-C: High density lipoprotein cholesterol; LDL-C: low-density lipoprotein cholesterol.

Supplementary Table S2: Outcome stratified by dose appropriateness

**Outcomes**		**Frequency**			**P-Value**
		**Inappropriate dose**	**Appropriate dose**	**Total**	
**Bleeding**		14(56%)	20(48.8%)	34(51.5%)	0.569
**Bleeding type**	Minor	11(44%)	13(31.7%)	24(36.4%)	0.584
	Major	3(12%)	7(17.1%)	10(15.2%)	
**Dose appropriateness**	Low	19(76%)	0(0%)	19(28.8%)	<0.001
	Appropriate	0(0%)	41(100%)	41(62.1%)	
	High	6(24%)	0(0%)	6(9.1%)	
**DVT/PE during follow up**		1(4%)	2(4.9%)	3(4.5%)	1
**Stroke during follow up**		0(0%)	3(7.3%)	3(4.5%)	0.283
**Mortality**		10(40%)	13(31.7%)	23(34.8%)	0.597
